# Insights into the Epidemiology, Pathogenesis, and Therapeutics of Nonalcoholic Fatty Liver Diseases

**DOI:** 10.1002/advs.201801585

**Published:** 2018-12-12

**Authors:** Yao Yu, Jingjing Cai, Zhigang She, Hongliang Li

**Affiliations:** ^1^ Department of Cardiology Renmin Hospital of Wuhan University Jiefang Road 238 Wuhan 430060 P. R. China; ^2^ Institute of Model Animal Wuhan University Donghu Road 115 Wuhan 430071 P. R. China

**Keywords:** diagnosis, epidemiology, nonalcoholic fatty liver disease (NAFLD), pathogenesis, treatments

## Abstract

Nonalcoholic fatty liver disease (NAFLD) is the most common liver disease which affects ≈25% of the adult population worldwide, placing a tremendous burden on human health. The disease spectrum ranges from simple steatosis to steatohepatitis, fibrosis, and ultimately, cirrhosis and carcinoma, which are becoming leading reasons for liver transplantation. NAFLD is a complex multifactorial disease involving myriad genetic, metabolic, and environmental factors; it is closely associated with insulin resistance, metabolic syndrome, obesity, diabetes, and many other diseases. Over the past few decades, countless studies focusing on the investigation of noninvasive diagnosis, pathogenesis, and therapeutics have revealed different aspects of the mechanism and progression of NAFLD. However, effective pharmaceuticals are still in development. Here, the current epidemiology, diagnosis, animal models, pathogenesis, and treatment strategies for NAFLD are comprehensively reviewed, emphasizing the outstanding breakthroughs in the above fields and promising medications in and beyond phase II.

## 1 Introduction

Nonalcoholic fatty liver disease (NAFLD) is becoming the most common chronic liver disease. It is defined as at least 5% steatosis observed in the hepatocytes on either histology or by imaging methods such as proton density fat fraction (PDFF),^1^, ^2^, ^3^, ^4^ with the absence of drug abuse and excess alcohol intake (i.e., within the threshold of <30 g d^−1^ for men and <20 g d^−1^ for women).^4^, ^5^, ^6^ NAFLD comprises a spectrum of liver diseases, ranging from simple steatosis through steatohepatitis (NASH, steatosis plus inflammation and ballooning degeneration) to advanced fibrosis, cirrhosis, and ultimately hepatocellular carcinoma (HCC). NASH is not always preceded by steatosis. Steatosis and NASH can be considered as completely separated entities, although it is generally conceived to be a sequential process from steatosis to NASH and fibrosis, and the progression from steatosis to NASH has been proven in many patients.^1^ NAFLD is tightly linked to metabolic disorders, such as metabolic syndrome (MetS), insulin resistance (IR), type 2 diabetes mellitus (T2DM), obesity, hyperinsulinemia, and cardiovascular diseases.^7^, ^8^, ^9^, ^10^, ^11^, ^12^, ^13^, ^14^ It is estimated that 25% of the global population struggles with NAFLD, and the prevalence is growing dramatically.^15^, ^16^ The prominent causes of death in NAFLD are cardiovascular diseases and extrahepatic malignancy, whereas less than 5% of NAFLD patients die from liver‐related factors.^17^, ^18^, ^19^ Inadequate awareness and the lack of effective noninvasive screening and diagnostic tools for the general population are the primary contributors to the morbidities associated with NAFLD.

**Figure 1:**
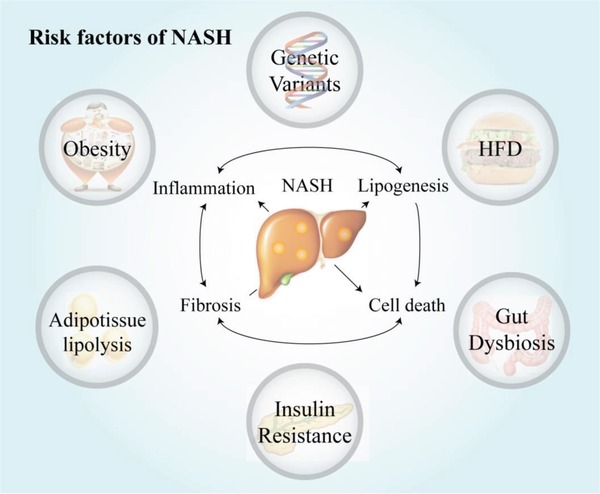
Risk factors of NAFLD. NAFLD is a complex and multifactorial disease; myriad of factors including environmental factors, gut microbiota, insulin resistance, obesity, as well as genetic and epigenetic factors are all implicated in the pathogenesis of the disease. HFD, high‐fat diet.

NAFLD is a complex and multisystemic disease, and its pathogenesis involves many factors, including genetic factors,^20^ environmental factors, and metabolic factors (**Figure** **1**).^21^, ^22^ The pathogenesis in various individuals might be discrepant, which increases the difficulty of exploring the pathogenesis of NAFLD. A major obstacle to the study of NAFLD pathogenesis is the lack of ideal animal models that imitate human NAFLD perfectly in both phenotype and mechanism. The most commonly used rodent animal models are genetically far removed from humans and cannot exactly represent human NAFLD; however, large animal models, such as primate models, can overcome the shortcomings of the mouse model.^23^ Although meaningful breakthroughs regarding the pathogenesis of NAFLD have been achieved in recent decades and therapeutics targets and medication development have been clarified, unmet challenges remain unavoidable. To date, the most frequently recommended way to improve NAFLD continues to be lifestyle modification, which is difficult to sustain long term, and effective approved drugs are still in development.^24^, ^25^ Both the serious health problems and economic factors contribute to the developments of the pharmaceuticals for NAFLD. More than 600 clinical trials are active on *clinicaltrials.gov*; their therapeutic targets are varied, ranging from metabolism‐related and anti‐inflammatory factors to antifibrotic targets, almost covering all the key regulators in the pathogenesis of NAFLD.^26^, ^27^, ^28^, ^29^

Further investigations of the diagnostic methods, especially the noninvasive ones, and pathogenesis, which can accelerate the medication development, and the development of ideal animal models are urgently needed to restrain this epidemic NAFLD. This review focuses on the current understanding of NAFLD, providing a critical summary of five areas including epidemiology, diagnosis, animal models, pathogenesis, and therapeutics, emphasizing the important breakthroughs and the most promising drugs in clinical trials as well as the emerging therapeutic targets, in hopes of bringing new insights into the characterization of NAFLD.

## 2 Epidemiology

NAFLD is highly prevalent worldwide and has spread rapidly along with the increase in sedentary lifestyles and the obesity epidemic, which result from the popularity of fast food and sugary beverages and from economic developments. Small‐scale investigations estimate that the global prevalence of NAFLD is 25%.^30^, ^31^, ^32^ The prevalence of NAFLD varies by region, ethnicity, age, and socio‐economic status.

In general, in terms of geographic location, the three regions with the highest rates of NAFLD are the Middle East, South America and Asia, where the prevalence are 32%, 31%, and 27%, respectively, followed by the well‐studied USA, where the prevalence is 25%.^15^ In Africa, NAFLD is less common, with a prevalence of 14%.^33^ An article reported that the average NAFLD prevalence is 23.71% in Europe, varying from 5% to 44% in different countries.^31^ The prevalence in northeastern Germany is ≈30%, as determined by ultrasonography, whereas the prevalence is 26.4% in the UK, as diagnosed by liver biochemistry markers.^34^ Liver biopsies determined the prevalence in France to be 26.8%, and 32.7% of this group had NASH.^15^ In the Asia‐Pacific region, the economic, political and educational levels and nutrition and lifestyle factors, contribute to the varying prevalence of NAFLD in different locations. Due to the development of the economy and health care system, the epidemiological data for this region are not as comprehensive as the data for more developed countries.^35^ However, the increasing prevalence of NAFLD is similar to the tendency observed worldwide. The prevalence of NAFLD in rural India, where people have traditional diets and lifestyles, is 9%, whereas in urban populations, the prevalence ranges from 16% to 32%.^36^ The same variances between rural and urban areas have been observed in other countries, such as Malaysia, Sri Lanka, Singapore and Indonesia. Detailed data regarding the nationwide prevalence of NAFLD in China are rare but existing studies have revealed that NAFLD is poised to become the leading liver disease, replacing viral liver disease.^37^, ^38^ Similar to other countries, the prevalence varies among different regions of China and is higher in urban areas than in rural areas.^39^ The prevalence in Shanghai and Guangdong are 15.0% and 17.0%, respectively.^40^, ^41^ The NAFLD prevalence in Hong Kong, determined by proton magnetic resonance spectroscopy (^1^H‐MRS), is 28.8%, and 60.5% of these patients are obese.^42^, ^43^

The prevalence of NAFLD also varies among different ethnic groups. It has been reported that NAFLD is most prevalent in Hispanic Americans, followed by European and African Americans, although obesity and hypertension are more prevalent in African Americans.^15^ NAFLD is more commonly observed in men than women, which might be associated with the sex hormones, and differences also depend on ages.^22^ Children are also affected by NAFLD; and ≈25% of young people in the USA currently suffer from this chronic disease, whereas the prevalence was only 9.6% in 1988–1994.^44^, ^45^, ^46^

The prevalence of NAFLD parallels that of T2DM, obesity and MetS. It is currently reported that 80% of NASH patients are obese, 44% have T2DM, and 72% are at risk of dyslipidemia.^31^ Individuals with these diseases are at even greater risk of NAFLD than the general population. Specifically, the reported prevalence of NAFLD is as high as 91% in the obese patients with a body mass index (BMI) ≥30 kg m^−2^, 67% in patients with a BMI of 25–30 kg m^−2^ patients and 25% in normal‐weight patients.^47^ Interestingly, there is a type of lean NAFLD found in nonobese NAFLD patients with few obesity‐related comorbidities.^4^, ^48^, ^49^ The mechanism of lean NAFLD is complex, and high‐fat and high‐fructose diets and genetic factors might all be effectors. Patients with lean NAFLD are always younger, frequently have sedentary lifestyles, and show impaired insulin sensitivity, high TG levels and high cardiovascular risk. However, the proportions of advanced fibrosis progressing from steatosis in the normal NAFLD and lean NAFLD are the same.

The exact number of prevalence might differ due to the various diagnosis methods employed. In the majority of current studies, NAFLD screening is based on imaging, such as ultrasonography, or on biochemical‐related methods, such as the fatty liver index, both of which are noninvasive methods with high acceptance. Because these methods are indirect, they have limited accuracy and credibility, which might be part of the reason why accurate epidemiological data are lacking. To further elucidate the nature of NAFLD, large‐scale, longitudinal population studies and reliable noninvasive screening tools are essential.

The morbidity and mortality directly related to liver pathological changes account for about 10% NAFLD patients while the leading cause of NAFLD is cardiovascular diseases (CVD) accounting for about 40–45% of the total death.^17^, ^18^, ^19^ Meanwhile, NAFLD is widely accepted as a systematic metabolic disease, which facilitates the development of CVD. It is reported that the prevalence of CVD is more than 40% among the patients with NAFLD.^12^, ^17^, ^19^

## 3 Diagnosis

Over the past few decades, tremendous achievements have been made in the evaluation of NAFLD to meet the urgent needs for screening and diagnosis of NAFLD.^50^ Noninvasive methods, such as imaging tools and serum biomarkers, have evolved greatly.^51^, ^52^, ^53^, ^54^, ^55^ Furthermore, various biomarker indices have been created to describe and diagnose NAFLD, and these are more accurate than single biomarkers.^56^, ^57^ However, biopsy is still the gold standard for the assessment of the severity of the disease, and it is the most accurate way to diagnose NAFLD based on histological results.^58^, ^59^

### 3.1 Noninvasive Imaging

Ultrasonography, magnetic resonance imaging (MRI), elastography, and computed tomography (CT) are the most common tools used to diagnose NAFLD clinically.^60^

Ultrasonography is currently the most widely used imaging tool in clinic and the most acceptable method for the first‐line screening of steatosis since it is easy to perform and less expensive than other advanced imaging methods.^4^ The mechanism is the increased echogenicity caused by the intracellular accumulation of lipid vesicles in hepatic steatosis. The sensitivity of ultrasonography is limited by the thickness of peripheral tissue, and when less than one third of the liver parenchyma is infiltrated by lipid droplets, the results are not reliable.

MRI has been widely used in clinical diagnosis recently because of its high sensitivity and because it is the only way to quantitate the liver fat.^4^ ^1^H‐MRS is a type of MRI that is used for the direct measurement of the chemical composition of tissue. In NAFLD, ^1^H‐MRS can be used to detect the spectral peaks of triglycerides. And it shows excellent accuracy and sensitivity, but has a high cost. ^1^H‐MRS is able to detect very low fat quantities and can be used to determine the prevalence of NAFLD in general populations.

Elastography is based on the principle of ultrasonography and can measure the stiffness of the liver. Four different types of devices are used for NAFLD: vibration control transient elastography (VCTE; FibroScan), acoustic radiation force impulse (ARFI), 2D shear wave elastography (2D SWE), and magnetic resonance elastography (MRE).^54^, ^61^ VCTE is the first FDA‐approved elastographic method and might be the most widely used method to date. In VCTE, a handheld probe is employed to introduce a mechanical shear wave to liver, and the wave propagation is detected and quantified. There are two different types of probes: XL probe is suitable for obese patients and M probe is used for the normal‐weight patients.^61^ VCTE shows excellent accuracy for the exclusion of the advanced fibrosis, but it is not very sensitive with a positive predictive value for the advanced fibrosis and cirrhosis. ARFI, also known as pSWE (point shear wave elastography), is considered to be a second‐generation elastographic diagnostic method, which can be easily enabled by modifying commercial ultrasonography machines. ARFI is more suitable for severe fibrosis and cirrhosis than for mild cases but the operator is a factor that influences the results. 2D‐SWE, similar to ARFI, can also be implemented on any commercially available ultrasonography machine and operator‐independent but more accurate than ARFI for the diagnosis of mild or stage F2 fibrosis. MRE, which combines MRI and elastography, shows great accuracy but is limited by its high cost and low availability. MRE is more accurate than VCTE in the diagnosis of F2 and F4 fibrosis, and its failure rate is much lower than those of the other tools discussed above. The iron burden in the liver can be a negative factor for the examination, as can obesity (a body weight > 160 kg).^60^ Because of the high cost of the advanced elastography, only the higher‐risk patients are recommended to undergo elastography examinations.^3^

CT can also be used for the diagnosis of fatty liver if attenuation is lower than 40 HU or if the CT value of the liver is 10 HU less than that of the spleen.^60^ Based on the mechanism of CT, fatty liver and lipid droplets, which are made of ‘light’ atoms, cannot be clearly observed. Moreover, the possible radiation exposure limits the application of CT in children and pregnant women and for longitudinal studies. **Table** **1** shows a detailed comparison of the noninvasive diagnostic imaging methods currently used for NAFLD.

**Table 1:** Comparison of imaging tools for the diagnosis of NAFLD. TAG, triglycerides; VCTE, vibration control transient elastography; ARFI, acoustic radiation force impulse; SWE, shear wave elastography; MRE, magnetic resonance elastography; CT, computed tomography; MRI, magnetic resonance imaging

Methods	Cost	Accuracy	Quantification	Sensitivity	Application	Limitation
**Ultrasonography**	+	+	No	Decrease with obesity	Screening tool, inexpensive	Operator dependent, low sensitivity,
**Elastography**						
VCTE	+	++	Yes	High	Specific for liver, fast acquisition, advanced fibrosis and cirrhosis, immediate results; XL probe for overweight patients	Disable to tell fibrosis stage, not reliable in severely obese patients
ARFI	++	++	Yes	High	Specific for liver, fast acquisition, advanced fibrosis and cirrhosis, immediate results	Operator dependent; limited data, narrow range
SWE	++	++	Yes	High		Operator dependent
MRE	+++	+++	Yes	High	Not affected by obesity	High cost, time consuming, not suitable for patients with implantable devices
**CT**	++	++	Semi	Low	/	Radiation exposure
**MRI**	+++	++	Yes	High	Gold standard for TAG evaluation	Operator dependent, long imaging time, limited availability

### 3.2 Serum Biomarkers

In addition to imaging methods, serum biomarkers are noninvasive tools for diagnosing NAFLD that are convenient and effective; and they can be used as diagnostic tools when imaging tools are not available. The different stages of NAFLD, including steatosis, inflammation and fibrosis, involve numerous hormonal and small molecular disturbances that can be detected in serum and used as biomarkers to diagnose and predict the disease progression. Aminotransaminase (ALT) is the most commonly used biomarker of chronic liver disease to evaluate the function of the liver.^57^ However, in NAFLD, the ALT level alone provides limited information and is poorly predictive because of its low specificity. According to the pathogenesis of liver injury in NASH, specific serum biomarkers can be used to diagnose NASH.^55^, ^56^ Hepatocyte death occurs via apoptosis or necroptosis increasing in NASH, resulting in the release of cytokeratin 18 (CK18), a type of intermediate filament protein. Using an antibody enzyme‐linked immunosorbent assay (ELISA), serum CK18 levels can be measured sensitively; however, their clinical application is premature.^26^, ^49^ Inflammation is also a histological hallmark of NASH, and many inflammatory markers and mediators could serve as biomarkers of NASH, including tumor necrosis factor (TNF), interleukin‐6 (IL‐6), IL‐8, and C‐reactive protein (CRP). Other biomarkers, such as the lipid oxidation products, could also be used for diagnosis because oxidative stress is an important pathogenic mechanism of NASH; however, measurement of these biomarkers is limited by its cost and the need for specialized equipment. Hormones related to lipid and glucose metabolism, including adipokines (leptin, resistin, and adiponectin) and fibroblast growth factor 21 (FGF21), can be biomarkers for NAFLD, but with low specificity since they are also associated with other disorders, including MetS.^56^ Serum biomarkers are easy to measure, but their low specificity and sensitivity have limited their wide application. Over the past few decades, especially before advanced imaging tools were available, scientists developed several indices based on serum biomarkers plus anthropometric parameters, such as weight, height and waistline, in hopes of increasing the accuracy and specificity of NAFLD diagnosis.^52^

For the diagnosis of steatosis, one of the best indices is the SteatoTest,^62^ a formula adapted from the FibroTest,^63^ which uses five variables: BMI, cholesterol, triglycerides (TAG) and glucose, adjusted by age and gender. The fatty liver index^64^ consists of waist circumference, BMI, γ‐glutamyltranspeptidase and TAG, while the NAFLD liver fat score^65^ utilizes fasting insulin level, AST level, the AST: ALT (alanine aminotransferase) ratio, T2DM, and MetS. Together, BMI, diabetes, and the AST: ALT ratio together are called the hepatic steatosis index.^66^ NAFLD ridge score is believed to have an excellent negative prediction ability to exclude NAFLD.^67^ None of these indices is widely used, and they have failed to reveal too much information in either clinical or laboratory studies. Similarly, various scoring systems have been developed to predict NASH and fibrosis. The FIB‐4 score^68^ was developed to identify advanced fibrosis in patients with comorbid human immunodeficiency virus (HIV) and hepatitis C virus (HCV). The BARD score^69^ was created to describe advanced fibrosis using several variants. The Hepascore,^70^ which employs six parameters, seems more accurate than the BARD score and the APRI in the diagnosis of F3‐4 fibrosis. Moreover, the NAFLD fibrosis score^71^ was created for the diagnosis of advanced fibrosis. In contrast to the scoring system for steatosis, these fibrosis indices are accurate, and some of them are even more accurate than the imaging results.^54^ The combination of elastography and indices or biomarkers can provide marked diagnostic accuracy, which could save many biopsies. However, these scores are not sufficient to replace liver biopsy in patients who are suspected of advanced fibrosis. **Table** **2** presents detailed information about the above indices for steatosis and fibrosis.

**Table 2:** Score systems for NAFLD. AUROC, area under receiver operating characteristics curve; BMI, body mass index; WC, waist circumference; TAG, triglyceride; GGT, gamma‐glutamyl transpeptidase; T2DM, type 2 diabetes mellitus; AST, aspartate transaminase; ALT, Alanine transaminase; DM, diabetes; MetS, Metabolism syndrome; HDL, high density lipoproteins; WBC, white blood cells; BG, fasting blood glucose, BIL, total bilirubin; CV mortality, cardiovascular mortality; HCV, hepatitis C virus

Score system	Description	Reproducibility	AUROC	Application	Limitation	Refs
***For steatosis***						
Fatty liver index	BMI, WC, TAG, GGT	+	0.84	High applicability and commonly used in clinical and laboratory	Modest accuracy, complex formula	64
The liver fat score	MetS, T2DM, fS‐insulin, fS‐AST, AST: ALT ratio	+	0.86–0.87			65
Hepatic steatosis index	BMI, AST: ALT ratio, gender, DM	+	0.81	Suboptimal gold standard	Cannot distinguish steatosis stage	66
SteatoTest	BMI, glucose, TAG, cholesterol, ALT, GGT	+	0.79–0.80	Sensitive, uncommon used	High cost, modest specificity	62
NAFLD ridge score	ALT, HDL‐C, TAG, WBC, hypertension, HbA_1c_	+	0.87	Limited to research setting	Low positive prediction values	67
***For fibrosis***						
FIB‐4	Age, AST, ALT, platelet count	Not tested	0.80 for F3 fibrosis	Predicts all cause and CV mortality, liver‐related events	Modest responsiveness	68
BARD score	AST, ALT, BMI, diabetes	Not tested	0.69–0.81 for F3 fibrosis	Predicts liver‐related events	BMI is various in different ethnic groups	69
NAFLD fibrosis score	Age, BMI, AST, ALT, platelet count, diabetes, albumin, BG	Not tested	0.84	Predicts liver‐related events, all cause and CV mortality	BMI interpretation not independent with ethnic group	71
HepaScore	Age, gender, HA, BIL, GGT, α2‐macroglobulin	NA	0.81	Developed in HCV	Cannot distinguish fibrosis stage	70
FibroTest	GGT, BIL, haptoglobin, apoAI and α2‐macroglobulin	+	0.81	Predicts overall mortality, accurate in obese patients	Suboptimal gold standard for early‐stage fibrosis	63

### 3.3 Genetic Biomarkers

Genetic biomarkers are a new type of noninvasive diagnostic method for NAFLD that are rarely used currently.^55^ With the development of genome‐wide association studies (GWASs) and high‐throughput technologies in the past few decades, the genetic factors of NAFLD has been well studied. Thus, the genetic biomarkers could be a useful strategy to screen individuals with hereditary susceptibility of NAFLD. Genetic biomarkers for NAFLD include DNA sequence variations, such as SNPs (single nucleotide polymorphisms), and miRNA (microRNA). The best‐studied SNPs in NAFLD currently are rs738409 and rs58542926, which are located in PNPLA3 and TM6SF2, respectively.^72^ Both these biomarkers have impacts on the phenotypes and histological outcomes of NAFLD. miRNA is also a hallmark of NAFLD that can reveal the dynamic changes, among which the best probe is miR‐122.^73^ However, the data showed that the use of genetic biomarkers failed to increase the accuracy of diagnosis of NAFL and NASH,^72^ indicating that substantial advancement is needed before genetic biomarkers are able to use in clinical testing.

### 3.4 Invasive Biopsy and Histological Score

Biopsy remains the gold standard for assessing the severity of NAFLD and the only method for the evaluation of inflammation in NAFLD, such as NASH. Biopsy cannot be substituted by the methods discussed above because of its accuracy and its ability to detect the disease grade and stage. However, biopsy is limited by its invasive features, morbidity and mortality risks and bias on the sampling and pathology.^3^, ^49^, ^59^ Thus, biopsy cannot be used as a screening method in large populations. Furthermore, due to the small size of the sample needle used in biopsy, the large livers might not be well represented by the tiny samples, which are only ≈1/50 000 of the volume of the whole liver in volume. Steatosis activity fibrosis (SAF) and the NAFLD activity score (NAS) were developed to stage the histology section results of liver biopsy samples.^58^, ^74^ The NAS score consists of single scores for steatosis, inflammation and hepatocyte ballooning to quantify disease activity, and it has been proven to be inaccurate diagnosing of NASH due to the various criteria used by different groups. Thus, a more accurate scoring system, SAF, was created by the Fatty Liver Inhibition of Progression (FLIP) group. SAF score includes ranks steatosis (S: 0–3), activity of ballooning and inflammation (A: 0–4) and fibrosis (F: 0–4). In one study, all of the patients with NASH showed A>2, proving that SAF is an excellent diagnostic scoring system.^75^, ^76^ The invasiveness, sampling variability, high cost and morbidity risks limit the widespread acceptance of liver biopsy; thus, some reviews and guidelines have concluded the urgent and necessary situation for the applications of biopsy.^46^, ^53^, ^59^, ^61^, ^77^, ^78^

Based on the discussion above, ultrasonography could be the first‐line screening method for these suspected NAFLD patients to diagnose steatosis and exclude other liver pathology. Simple noninvasive markers of fibrosis, or score system or advanced imaging tools such as elastrography could be taken in confirmed steatosis patients to investigate fibrosis. However, these noninvasive methods show modest positive diagnosis value and only the biopsy can finally reveal the stage of NAFLD accurately.^50^, ^61^

Recent decades have witnessed great progress in the noninvasive evaluation methods for NAFLD, including imaging and serum biomarkers, which can serve as screening tools for the general population. However, biopsy cannot be replaced because noninvasive methods are not as accurate or reliable.

## 4 Animal Models

Ideal preclinical models that can mimic the biology and outcomes of human NAFLD are urgently needed to increase understanding of the pathogenesis and treatments for NAFLD.^75^, ^79^, ^80^ In the past few years, many animal models have been developed; especially mouse models obtained via dietary induction and genetic manipulations since mice are relatively inexpensive and accessible.^23^, ^81^ Large animals, such as primates, are reported to be ideal models for NAFLD as they resemble human more closely than rodent models do. It is imperative to recognize the drawbacks and limitations of these animal models before translating into practice when developing therapeutics (**Table** **3**).

**Table 3:** Animal models for the NAFLD. “+” means with the phenotype, “−” means without the phenotype. IR, insulin resistance; MCD, methionine‐ and choline‐deficient diet; CDAA, choline‐deficient L amino‐defined diet

Method	Phenotype						Advantages	Disadvantages
	IR	Obesity		Steatosis	Steatohepatitis	Fibrosis		
Diet‐induced								
High fructose	+		+	+	−	−	Includes MetS features	No spontaneous inflammation and fibrosis
High fat	+		+	+	+	+	Mimic human NAFLD	/
MCD	−		Weight loss	+	+	+	Imitate human NASH	Lose weight/cachectic, without features of MetS
CDAA	−		Weight loss	+	+	+	Best to mimic human NAFLD	Not mimic the pathogenesis of human NAFLD
Genetic manipulations								
ob/ob mouse	+		+	+	−	−	Includes MetS features	Need a second hit to NASH and fibrosis
db/db mouse	Glucose intolerence		+	+	−	−		

### 4.1 Genetically Modified Mouse Model

With developments in genetic engineering, it has become possible to generate different genetically modified animals. In NAFLD, the most commonly used genetic mouse models are ob/ob mice (leptin deficient) and db/db mice (leptin receptor deficient). Leptin, a type of hormone secreted by adipocytes in white adipose tissue, is very important in energy balance.^82^, ^83^ The ob/ob mouse shows a leptin deficiency because of a spontaneous mutation in the leptin gene. The lack of leptin function leads to the redistribution of fat from the adipose tissue to the liver, resulting in hepatocytes lipotoxicity. Therefore, these animals are indolent, hyperphagic, diabetic and have hyperglycemia and severe IR. However, the main difference between ob/ob mouse model and human NAFLD is that the ob/ob mouse cannot progress from steatosis to steatohepatitis spontaneously only if additional stimuli are offered. NASH is rarely observed in ob/ob mice because leptin is necessary for the activation of hepatic stellate cells (HSCs). The ob/ob mouse is protected from fibrosis, and ob gene mutations are not commonly observed in humans; moreover, leptin levels in serum are not correlated with NAFLD. Thus, the ob/ob mouse model has shown limited application in NASH. The db gene mutation results in leptin receptor deficiency, and the db/db mouse is similar to the ob/ob mouse in phenotype; it has normal leptin levels but is resistant to the effects of leptin. Hyperphagia, obesity, IR, hyperglycemia, hyperinsulinemia and fatty liver can be observed in the db/db mouse. A second hit, such as a high‐fat diet, is also needed to induce NASH or fibrosis in db/db mice. Furthermore, foz/foz mice, which are deficient in the Alms1 gene, sterol regulatory element‐binding protein 1 (SREBP1‐c) transgenic mouse, are all at the forefront of the exploration of the pathogenesis of NAFLD.

### 4.2 Diet‐Induced Mouse Model

Diets of different types of high‐calorie foods are key triggers of many diseases, such as MetS, NAFLD and cancers, and can also be used as an additional hit for disease progression in the genetic animal models. The majority of preclinical animal models of NAFLD are diet induced, such as with high glucose/fructose/sucrose, high fat, methionine‐ and choline‐deficient diet (MCD), high‐cholesterol diet (HCD), and choline‐deficient L amino‐defined (CDAA) diet. These diets are able to induce simple steatosis as well as steatohepatitis and advanced fibrosis.

Fructose consumption, which primarily comes from the corn syrup in soft drinks, is strongly linked with the progression of NAFLD because fructose can contribute to de novo lipogenesis.^84^, ^85^ The addition of fructose intake can lead to the hepatic TAG accumulation, steatosis, and obesity in 8 weeks and the intestinal bacterial overgrowth resulting in increased endotoxin levels and inflammation.^86^ Further animal data on the influence of fructose on metabolism‐related parameters are still needed. Animal models with high‐fat diets are created to imitate the Western lifestyle for the study of NAFLD. Compared with the MCD diet, high‐fat diets lead to much milder liver injury and result in IR and overweight. Lipid accumulation, oxidative stress, and abnormal mitochondria are also commonly observed in this model. A high‐fat diet can induce a model with metabolic parameters similar to those of human NAFLD but less severe hepatic pathology results.^87^ The MCD diet‐induced model is thought to be the most reliable model of the NAFLD spectrum regarding inflammation and fibrosis. Compared to other models, the MCD diet induces more oxidative products, causing oxidative stress, mitochondrial DNA damage and ultimately cell death. Thus, the MCD mouse shows inflammation and fibrosis in addition to TAG accumulation in the liver. However, the most essential difference between this model and NAFLD in humans is that the MCD mouse model is always cachectic and lacks the IR in phenotype.^88^ A diet with high cholate‐ and cholesterol is used to induce steatosis, inflammation and fibrosis in 6–24 weeks in animals for the study of NAFLD. Hyperinsulinemia, obesity and the accumulation of hepatic free cholesterol (FC) can be found in mice, revealing the progression from NAFL to NASH. The CDAA diet‐induced model shows features similar to those of the MCD diet model, including steatosis, fibrosis, and cirrhosis but it does not exhibit severe cachexia features.^87^ Moreover, the CDAA mouse model can also develop IR in peripheral tissue and increased body weight, which are basic features of NAFLD. Thus, the CDAA model closely resembles human NAFLD progression but is not related to human NAFLD pathogenesis. The DIAMOND (diet‐induced animal model of NAFLD) has overcome some limitations of other models, meeting many of the requirements of a relevant model of human NAFLD. The DIAMOND shows IR, dyslipidemia, obesity, steatosis, inflammation, fibrosis and even spontaneous HCC. The main differences between DIAMOND and human NAFLD are the high HCC development rate, the decrease in cholesterol synthesis and the stepwise progression of NAFLD.^87^

### 4.3 Other Animal Models

Rodent models of NAFLD have been well developed and used for their advantages in cost and maneuverability, but large animal models seem to have more similarities to human NAFLD and greater possibilities for the experimental treatments in ahead of clinical trials. Ossabaw miniature swine can mimic human NAFLD excellently with a MetS and liver dysfunction in phenotype obtained via diet‐induced methods.

Nonhuman primates, such as monkeys, are employed as a NAFLD models in many studies since they have greater similarities with humans in anatomical structure, pathophysiological features and genetic profiles.^89^, ^90^, ^91^, ^92^, ^93^, ^94^ Specifically, monkey predisposed to MetS (MetS‐predisposed) could be an excellent model for NAFLD, which exhibits a higher body weight than healthy individual. MetS‐predisposed monkeys can develop obesity, dyslipidemia, and hyperglycemia spontaneously; additionally, mild NASH can occur early even when fed with a normal chow diet, and can progress to a severe NASH when fed with a high‐fat diet. In addition, the serum parameters of MetS‐predisposed monkeys change in parallel to the stage of cirrhosis, the same way they change in humans.^95^ All the features discussed above make MetS‐predisposed monkeys more suitable surrogates for human NAFLD than rodent models and may bring new insights into the pathogenesis of NAFLD. However, the difficulties to maintain large animals, the high cost, and the unsophisticated genetic modification method are all factors limiting the extensive applications of large animal models in NAFLD.^94^ Other animal species, such as zebrafish and fruit flies, have also been studied as the models for NAFLD, but the logistical difficulties, inherent differences and translatability challenges have limited their development and application.^96^, ^97^

## 5 Pathogenesis

**Figure 2:**
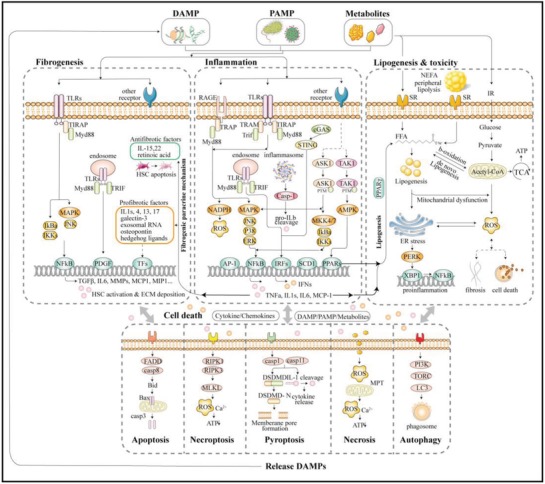
Signaling involved in the inflammation, metabolism, cell death, and fibrogenesis process of NAFLD. DAMP, danger‐associated molecular patterns; PAMP, pathogen‐associated molecular patterns; TLRs, Toll‐like receptors; RAGE, receptor for advanced glycation end‐products; SR, scavenger receptor; TIRAP, TIR domain‐containing adaptor protein; TRAM, TRIF‐related adaptor molecule; MyD88, myeloid differentiation factor 88; TRIF, TIR‐domain‐containing adaptor‐inducing interferon‐β; MAPK, mitogen‐activated protein kinase; JNK, Jun N‐terminal kinase; ERK, extracellular signal‐regulated kinase; IRF, interferon regulatory factor; MKK, MAPK kinase; ASK1, apoptosis signal‐regulating kinase 1; TAK1, TGFβ‐activated kinase 1; PPAR, peroxisome proliferator‐activated receptors; AMPK, 5′ adenosine monophosphate‐activated protein kinase; IFN, interferon; ER, endoplasmic reticulum; FFA, free fatty acids; ROS, reactive oxygen species; TCA, tricarboxylic acid cycle; PERK, protein kinase R‐like ER kinase; XBP1, X‐box‐binding protein 1; TNF, tumor necrosis factor; IL, interleukin; TFs, transcription factors; PDGF, platelet‐derived growth factor; MCP‐1, monocyte chemoattractant protein‐1; MMP, matrix metalloproteinase; MLKL, mixed lineage kinase domain‐like protein; RIPK, receptor‐interacting proteins kinase; MPT, mitochondrial permeability transition.

NAFLD is a complex disease, and its pathogenic drivers and clinical manifestations are highly heterogeneous among various individuals.^98^, ^99^, ^100^ A ‘two‐hit’ theory was proposed in 1998 to describe the pathogenesis of NAFLD; it proposed that steatosis is the first hit for NAFL, and a second hit, such as oxidative stress, is needed for the progression to NASH and advanced fibrosis.^101^ However, this hypothesis was too simple and outdated. A multiple‐hit hypothesis involving a myriad of factors offers a more acceptable delineation of the pathogenesis of NAFLD.^102^ Our lab has made considerable achievements in identifying the pathogenesis of NAFLD and has revealed key molecular targets which could be potentially act as effective drug targets for NAFLD.^103^, ^104^ In this section, the mechanisms are discussed on the view of inflammation, metabolic homeostasis, fibrosis and genetic factors related to NAFLD (**Figure** **2**).

### 5.1 Inflammation and Innate Immunity

Inflammation is the prominent hallmark of NASH and can be considered as the driving force of NAFLD. Inflammation in the liver can be triggered by many factors, such as gut microbiota, metabolic disorders, and genetic and epigenetic factors. Hepatitis can be triggered by the activation of immune cells, resulting in tissue damage which lays a foundation for advanced fibrosis and cirrhosis.^105^ Furthermore, inflammation may induce lipid accumulation, and in NASH, inflammation can precede steatosis.^102^ Hepatocyte injury, cell death, and inflammation can occur at very early stages of diseases and metabolic disorders including steatosis, are considered to be catalyzed by mild inflammation in the hepatocytes.^106^ Thus, exploring the complexity of the inflammation and innate immunity can improve our understanding of the mechanism underlying NAFLD and aid in the development of the novel therapeutics.^103^

The abundant blood supply and unique sinusoid microstructure place the liver at the frontline in immune responses, and its profound functions provide a significant barrier in the development of immune responses.^107^ The blood supplied to the liver is enriched in pathogen‐associated molecular patterns (PAMPs), microbe‐associated molecular patterns (MAMPs), and the danger‐associated molecular patterns (DAMPs).^108^, ^109^ Microbiota dysbiosis and intestinal permeation disorders, which are always a feature of MetS, induce plentiful gut‐derived products translocated into portal circulation, provoking the immune responses and chronic inflammation in the liver via PAMPs. The best‐studied bacterial product is the bacterial endotoxin (lipopolysaccharide, LPS) found from the outer membrane of gram‐negative bacteria. LPS can activate immune cells via Toll‐like receptors 4 (TLR4), which is a pattern‐recognition receptor (PRR) expressed on the membrane of macrophages and other sentinel cells. One of the downstream pathways is myeloid differentiation factor 88 (MyD88)‐dependent which is linked with the translocation of nuclear factor‐κB (NF‐κB), resulting in the release of proinflammatory cytokines including tumor necrosis factors α (TNFα) and interleukin‐1β (IL‐1β). In addition to MyD88, the other adaptors of TLRs are TIR‐domain‐containing adaptor‐inducing interferon‐β (TRIF), TIR domain‐containing adaptor protein (TIRAP) and TRIF‐related adaptor molecule (TRAM). Furthermore, the TLRs pathway comprise of MyD88‐dependent pathway and TRIF‐dependent pathway. TRIF is often regulated when MyD88 is deficient in the liver whereas both of the two pathways are important in the activation of IRF related molecular events. For TLR2/4, MyD88 adaptor‐like protein (MAL, or TIRAP) is essential for the recruitment of MyD88 to the Toll and IL‐1 receptor (TIR) domain of receptor.^110^ Our previous works have revealed the importance of TLR4 and its related pathways in the progression of NAFLD, suggesting that these could be utilized as promising medical targets.^90^, ^111^, ^112^ For other TLRs, such as TLR9 acting with unmethylated CpG motifs and TLR2 acting with peptidoglycan, more data are needed to determine their functions in the immune response and progression of NAFLD. TLR9 is reported to be closely associated with the activation of HSCs and fibrogenesis via the TLR9‐MyD88 pathway.^113^ Furthermore, TLR9 is also implicated as a sensor of free genomic double‐stranded DNA (dsDNA) released during the apoptosis, a cell death process.^114^ TLRs can also located on the endosome and they can regulate the downstream molecules including interferon regulatory factors (IRFs) and NF‐κB via MyD88‐ and TRIF‐dependent pathway.^109^, ^115^

Oligoadenylate synthase (OAS)‐like receptors (OLRs) are a type of fresh identified PRRs for the nucleic acids which include OAS homolog cGMP‐AMP synthase (cGAS).^103^ Cytosolic dsDNA, including microbial DNA and self DNA, can induce an immune response via DAMPs and active stimulator of interferon genes (STING) via cGAS, which are crucial for cell death in vitro. Cellular FLICE‐inhibitory protein (cFLIP) was reported to be associated within this inflammation and the immune response process and can act as a drug target for NASH.^114^, ^116^ Moreover, another PRR, cytoplasmic nucleotide‐binding oligomerization domain (NOD)‐like receptors (NLRs), is involved in a wide range of immune and cell death pathways. The best‐studied NLRs, NLRP3, can form inflammasomes in addition to adaptor proteins and serine protease caspase‐1, which is a key mediator in cell death. Inflammasome activation in hepatocytes leads to the release of proinflammatory cytokines and increased apoptosis. Advanced glycation end‐products (AGEs) or glucotoxins, which are enriched in high temperature‐cooked food including fried, baked or broiled food and western diets, are obtained from the carbohydrates reacting with proteins nonenzymatically. The typical receptors for AGEs, the receptor for AGEs (RAGEs or AGERs) which is also a type of PRRs, are highly expressed on inflamed areas and hepatic stellate cells (HSCs) which play important roles in the process of inflammation and fibrosis. The activation of RAGE‐ NF‐κB and RAGE‐MAPK pathways are commonly detected in NASH patients.

After the stimulation of PRRs, innate immune cells are activated, and subsequently, a cascade of immune reaction implicated with many signal pathways and mediators are triggered. Members of the mitogen‐activated protein kinase (MAPK) families, including the p38, Jun N‐terminal kinase (JNK) and extracellular signal‐regulated kinase (ERK); and the upstream MAP3Ks, such as apoptosis signal‐regulating kinase 1 (ASK1) and TGFβ‐activated kinase 1 (TAK1); and the transcription factors, including NF‐κB and interferon regulatory factors (IRFs) together regulate the inflammatory responses in hepatocytes.^110^ Moreover, TNF receptor‐associated factors (TRAFs), which can regulate the MAPK and NF‐κB pathways via post‐translational modifications (PTMs), also play prominent roles in the progression of NAFLD; in particular, TRAF5 is reported to be a negative regulator of hepatic steatosis.^117^, ^118^, ^119^ ASK1 is apical kinases in the MAPK pathways regulating the NF‐κB pathway, mediated by p38 and JNK as well as MAPK kinase 4 (MKK4) and MKK7 while TAK1 can modulate PPARs via 5' adenosine monophosphate‐activated protein kinase (AMPK) which is an important regulator in lipid metabolism. It has been reported that ASK1 activation is a key process in the progression of NAFLD, and ASK1 inhibitors are among the hottest drug targets.^120^ TRAF1 and TRAF6 can be utilized to promote the activation of ASK1 whereas deubiquitinase TNF‐a‐induced protein 3 (TNFAIP3), dickkopf‐3 (DKK3), CASP8 and FADD‐like apoptosis regulator (CFLAR), cellular repressor of E1A‐stimulated genes (CREG) and caspase recruitment domain 6 can suppress ASK1 activation, which improves the NASH.^89^, ^91^, ^121^, ^122^, ^123^, ^124^, ^125^, ^126^, ^127^ Similarly, hyperactivation of TAK1 can also exacerbate NASH, and impaired activation of TAK1 via deubiqutination or dephosphorylation could be good strategies to treat NASH. Cylindromatosis (CYLD), ubiquitin‐specific protease 18 (USP18), and dual‐specificity phosphatase 14 (DUSP14) are reported to be candidate enzymes for suppressing the progression of NASH via inhibiting TAK1,^92^, ^128^, ^129^, ^130^ whereas the E3 ligase tripartite motif 8, TRAF3, promotes NASH.^128^, ^131^, ^132^, ^133^ Transcription factors are at the downstream of the intracellular signal pathway and can modulate the expression of effectors that influence the pathogenesis of NAFLD. IRFs are recognized to work in the progression of NAFLD in addition to their original functions which are the regulation of the expression of type I IFN and IFN‐induced genes.^111^, ^115^, ^134^, ^135^, ^136^ Different types of IRFs play various roles. IRF3 is proved to alleviate the lipid accumulation and to improve IR in the liver, and IRF9 can be used to ameliorate steatosis and inflammation.^137^, ^138^, ^139^, ^140^, ^141^ NF‐κB and its regulator IκB kinases (IKKs) are implicated in inflammatory responses and metabolism. Leukocyte immunoglobulin‐like receptor B4 deficiency was reported to increase inflammation via NF‐κB signaling.^142^ And the inhibition of IKKε or TANK‐binding 1 kinase (TBK1) which is a noncanonical IKK family member, can improve steatosis markedly.^143^, ^144^

The gut‐liver axis was proposed as an important factor in the pathogenesis of NAFLD. The gut microbiota is involved in metabolic functions, vitamin synthesis and resistance to pathogens colonization in intestine. Dysbiosis, which is defined as a quantitative or qualitative modification of gut microbiota, is proved to be a predisposing factor for NAFLD. Translocation of bacteria or their products into the circulation is a prominent cause for dysbiosis contributing to NAFLD. Dysbiosis can change the permeability of the intestine, causing the microbiota or their products, such as LPS or dsDNA, to enter the portal circulation, which leads to inflammation and an innate immune response via the activation of JNK or NF‐κB signaling pathway and the release of proinflammatory cytokines, as discussed above.^145^, ^146^, ^147^, ^148^, ^149^ In addition, short‐chain fatty acids (SCFAs), including propionic, butyric and acetic acids, are the main products of carbohydrates digested by gut bacteria. These SCFAs are also involved in inflammation, the immune response process and the metabolism.^147^ The diversity and amount of SCFAs are dependent on the amounts of carbohydrates and dysbiosis. NAFLD patients have a less‐complex gut microbiome system than that of healthy individuals,^148^ and the balance of various SCFAs can identify their influence on intestinal permeability and inflammation.^147^

In addition to detecting the extraneous substances, the innate immune system can also sense the metabolic stress to induce inflammation via post‐translational modification or transcriptional regulation, which results in the promotion of metabolic disturbances that contribute to the progression of NAFLD.^106^, ^150^ In contrast, the metabolic states of immune cells during homeostasis and inflammation process are also crucial. The energy metabolism and specific activation of immune cells affect their proliferation, differentiation, and function. Thus, the manipulation of immunometabolism is burgeoning to be a potential therapeutic method for NAFLD.^106^

Inflammation is the key driver of NAFLD and all the PRRs, adaptors, kinases, transcription factors and their regulators, and gut‐related products involved in the inflammatory and innate immune responses can be acted as effective targets for the treatment of NAFLD.

### 5.2 Metabolic Homeostasis

It is normally accepted that steatosis is the mildest type of NAFLD that can be caused by the excess accumulation of lipids, fructose or other carbohydrates. Therefore, it is essential to clarify the source and fates of the energy resources, such as free fatty acids (FFAs) and carbohydrates in the liver; if the supply is overloaded, or the disposal is disrupted, the excess energy substance can be toxic, causing endoplasmic reticulum (ER)/oxidative stress and cell injury.^124^, ^151^, ^152^

The accumulation of fat, mainly triglycerides, within the hepatocytes during the fasting state derives from three principal sources: ≈15% from the diet, ≈59% from the peripheral lipolysis or the none‐esterified fatty acid (NEFA) pool, and the remaining 26% from *de novo* lipogenesis (DNL).^21^ Lipids from the diets are primarily absorbed in the intestinal lumen, and then the bile acids (BAs) excreted by the hepatocytes hydrolyze the lipids to form nascent chylomicrons that are released into the circulatory system via the lymphatic system. Nascent chylomicrons are involved in the hydrolysis of TAG into fatty acids (FAs), which are finally taken up by the adipocytes and liver.^153^ FFAs in the liver can also arise from the NEFA pool. TAG in adipose tissue can switch to FFAs, which are delivered from the blood to the liver as a source of FFAs in the liver, which is mediated by the insulin. The fate of FFAs in the hepatocytes is also variable. Some FFAs can be oxidized in the mitochondria to create energy, and the majority can be converted into TAG, which can be stored in the hepatocytes or adipocytes as lipid droplets. Some FFAs can linked to the lipoproteins to form VLDL (very low‐density lipoprotein) or to synthesize phospholipids. Once FFAs homeostasis is impaired, the overload of FFAs leads to steatosis and lipotoxicity. In NAFLD patients, mitochondrial dysfunction and β‐oxidation disabilities are commonly observed, and lipids are stored in the liver in the form of TAG, which is a protective and adaptive response to lipid overload.

Dietary carbohydrates can also influence the FA homeostasis via DNL, and DNL is markedly increased in NAFLD patients.^154^ After meals, glucose is transported to the liver from the portal vein, and insulin regulated glycolysis occurs to decrease the glucose levels in the blood. Glucose can be metabolized into pyruvate via the glycolysis process, in which pyruvate kinase is a key enzyme, and decarboxylation can transform pyruvate to acetyl‐CoA, which is then utilized in the Krebs cycles or processed in the DNL process. With the regulation of insulin, extra glucose is normally stored as glycogen, or it can be esterified to TAG or VLDL via the DNL process.^84^, ^85^ In contrast, with glucose, which is highly regulated by insulin, almost all fructose can be transformed into FAs via DNL,^86^ which is precisely why high‐fructose diets can easily induce NAFLD.

IR, characterized by low sensitivity to insulin and the low glucose disposal in muscle and adipose tissue, is another important risk factor for the development and progression of NAFLD.^131^, ^138^, ^155^ IR has a direct effect on the metabolism of glucose which results in the lipid accumulation via DNL progress and in adipocytes, IR leads to the excessive delivery of FFAs to the liver.^117^, ^119^, ^156^ These above two processes both contribute to the extra lipid accumulation in the liver which could be a start of steatosis. Adipocytokines such as adiponectin, resistin and leptin, which are secreted by adipocytes, play key roles in regulating insulin sensitivity. In obesity, insulin‐mediated lipolysis in adipocytes is impaired, leading to an increase in NEFA, which interferes with insulin to affect glucose intake. Peripheral IR and hyperinsulinemia occur at this time, resulting in extra lipid delivery to the liver. Furthermore, IR is always linked to chronic mild inflammation, and lots of regulators released by adipocytes or immune cells can in turn promote IR, including TNFα, IL‐6, IL‐1 and monocyte chemoattractant protein‐1 (MCP‐1, also known as CCL2) as well as the IKK/NF‐κB pathway.^8^, ^157^, ^158^ Steatosis, inflammation which are two important progresses of NAFLD are both linked with IR closely. Thus, IR could be a good point to conquer the NAFLD.

### 5.3 Lipotoxicity

The overloaded TAG storage in hepatocytes exerts considerable stress on metabolism and subsequently causes lipotoxicity, which results in oxidative stress and ER stress.

β‐oxidation in the mitochondria is the main energy resource for the cells, and lipids are need to be transported from the cytoplasm into the mitochondria for oxidation. In general, the oxidation of very long‐chain fatty acids (VLCFAs) occurs in the peroxisomes, whereas other FAs are oxidized in the mitochondria via simple diffusion or with the help of the carnitine palmitoyl transferase 1. Oxidative stress is defined as reactive oxygen species (ROS) production that exceeds the ability of antioxidants. Generally, FFA can be oxidized in three different ways: α‐, β‐ and ω‐oxidation. Mitochondrial β‐oxidation and peroxisomal α‐, β‐oxidation happened in normal physiological condition. And when these above two ways are impaired, ω‐oxidation is believed to be an important rescue way.^159^ When FFAs are overloaded, the β‐oxidation in the peroxisome and the ω‐oxidation in the ER tend to produce ROS in hepatocytes. Kupffer cells is a main source of ROS via NADPH oxidase in the liver. It was observed that these two oxidation processes are increased in NAFLD patients, leading to ROS genesis and inhibiting mitochondrial β‐oxidation.^160^ Oxidative stress causes DNA damage in both nuclei and mitochondria and the release of cytokines related to inflammation and the membrane disruption.

The ER is an important organelle and a membranous network for the synthesis and assembly of biomacromolecules such as lipids, proteins and saccharides.^161^, ^162^ When FFAs are overloaded, the unfolded protein response (UPR) occurs in the ER. The UPR is an adaptive response to maintain homeostasis, and if the response fails, apoptosis occurs via stress‐sensor proteins, such as protein kinase R‐like ER kinase (PERK), activating transcription factor 6 and inositol‐requiring enzyme‐1 (IRE‐1). IRE‐1 is involved in the production of transcription factor X‐box‐binding protein 1 (XBP1), which interacts with inflammation via the JNK and IKK/NF‐κB signaling pathways.^114^, ^132^, ^133^, ^142^, ^162^

### 5.4 Cell Death

Periodic hypoxia, lipotoxicity, inflammasome activation, dysregulation of adipokines and cytokines, and products of the gut microbiome can all be factors in hepatocyte injury and death. Different forms of cell death, including apoptosis, necroptosis, pyroptosis, necrosis, and autophagy, play various roles in the progression of NAFLD.^153^

Apoptosis, one of the best‐defined types of programmed cell death, is considered a bridge between lipotoxicity and fibrogenesis, which acts as the main features in the NASH. Excess FFAs in hepatocytes induce mitochondrial dysfunction and lysosomal membrane permeabilization, resulting in apoptosis. In detail, the formation of FADD‐caspase 8 complex activates the proapoptotic protein Bid and the related mitochondrial pathway including the Bcl2 associated x protein (Bax) and generate more effector caspases to form apoptosome.^153^, ^163^ Apoptotic bodies and DNA fragments are important triggers for the activation of HSCs, leading to fibrogenesis.^140^, ^153^, ^164^ Furthermore, apoptotic signaling especially with death receptors such as TNFSF10, can induce inflammation and immune cell activation via the release of chemokines.^118^ Necroptosis is also a type of programmed cell death induced by receptors but caspase independent. The mediators of necroptosis are receptor‐interacting proteins kinase 1 and 3 (RIPK1, RIPK3) and mixed lineage kinase domain‐like protein (MLKL), associated with mitochondrial ROS production and Ca^2+^ leaking.^150^, ^165^ Pyroptosis is a caspase‐1‐dependent programmed form of cell death. Caspase‐1 activation via the NLRP3 inflammasome occurs in both macrophages and hepatocytes in the liver. Pyroptosis forms pores on the cell membrane, followed by the release of the cytoplasmic contents. NLRP3 was reported to be associated with collagen deposition and HSC activation and with the liver inflammation pathway via the cleavage of pro‐inflammatory cytokines and the release of IL‐18 and IL‐1β.^166^ Different to the programmed cell death forms discussed above, necrosis (oncosis) is a type of accidental cell death form. ATP depletion, ROS overload as well as drug‐ and toxin‐induced liver disease can all contribute to necrosis. Toxic stimuli can cause two different models of cell death based on the severity of insult, with low concentrations leading to apoptosis and the high concentrations inducing necrosis. It's reported that necrosis is regulated by mitochondrial permeability transition (MPT) which is implicated with cyclophilin D‐dependent MPT pore opening.^165^ Furthermore, autophagy is a dynamic process that controls the homeostatic functions of cells. Autophagy can remove damaged proteins and organelles, such as mitochondria, to protect the cell from dysfunction and death, whereas the attenuation of autophagy leads to oxidative stress or the release of mitochondrial factors triggering apoptosis.^153^, ^167^ The formation of autophagosome is regulated by mTOR and the antiapoptotic protein BCL2. Moreover, autophagy can activate the HSCs, although the mechanism is not yet clear.

Different forms of cell death can exert various impacts in NAFLD, and it should be emphasized that cell death occurs in the early stages of the disease and is closely linked with inflammation and fibrosis.

### 5.5 Fibrogenesis

A wound‐healing response in the liver leads to the genesis of liver scarring due to the deposition of high‐density extracellular matrix (ECM) proteins. In NAFLD, HSCs are crucial mediators of the fibrogenesis process.^168^ HSCs are normally used for the storage of vitamin A. The activation of HSCs induces complex events mediated especially by transforming growth factor β1 (TGF‐β1) and platelet‐derived growth factor (PDGF). These events are related to many types of peripheral cells, including Kupffer cells and macrophages and platelets. After activation, HSCs cannot store retinoid anymore; instead, they produce ECM components. Primary collagen deposition occurs in zone 3 of the acinus and then spreads to other areas, which are ultimately all bundled by collagen and progress to liver failure and HCC.^150^, ^169^ Excellent reviews of the mechanisms of fibrogenesis can be found elsewhere.^150^, ^168^, ^169^ HSCs also play an important role in inflammatory and immune responses in the liver. TLR4 is expressed on HSCs, and activated HSCs are highly sensitive to LPS via a TLR4‐dependent pathway, which leads to the release of cytokines such as IL‐8, resulting in the activation of the NF‐κB and JNK pathway.^147^ Many of the IKK/NF‐κB pathway related proteins are major player in the fibrogenesis progression of NAFLD, including MCP1 which is chemokine for monocyte recruitment, MIP1α which is applied for the leukocyte recruitment, MIP2 for the neutrophil recruitment, matrix metalloproteinase (MMP) which could degrade ECM and IL6 which is cytokines for the lymphocyte differentiation.^170^, ^171^

The past few decades have witnessed burgeoning developments in the pathogenesis of NAFLD, and considerable achievements have been made. However, the heterogeneous biology of NAFLD has not been entirely determined. Further investigations are needed to reveal the mechanisms more deeply, and potential medication/therapeutic targets are eagerly awaited.

### 5.6 Genetics

Plentiful of studies have approved that hepatic fat is strongly inheritable based on genetic and epidemiological methods.^172^ With the development of GWAS, the genetic factors linked to the progression and outcomes of NAFLD have become better known and have provided new possibilities for both the pathogenesis of and therapeutics of NAFLD.^20^, ^21^, ^173^, ^174^, ^175^ The best‐known candidate genes are PNPLA3 (patatin‐like phospholipase domain‐containing protein 3) and TM6SF2 (transmembrane 6 superfamily member 2).

PNPLA3 is located on chromosome 22 and encodes a protein of 481 amino acids that is related to the TAG hydrolase and PNPLA2 (adipose triglyceride lipase), associated with NAFLD in both steatohepatitis and fibrosis. PNPLA3 is highly expressed in human HSCs mediating retinol metabolism, particularly the storage of retinol. Many studies have reported the SNPs of PNPLA3; the most robust SNP of PNPLA3 is I148M, which has a variant at position 148 (rs738409 C>G) of methionine substituted from isoleucine. In in vitro experiments, the I148M group developed lipid droplets in hepatocytes and HSCs activated, while the wild type exhibited hydrolase activity for retinyl esters and TAG. The lipid accumulation in the I148 variant might cause cell injury and start the fibrosis process. However, there are conflicting results showing that I148M induced increased activity of lysophosphatidic acid acetyltransferase, thus increasing the synthesis of TAG.^176^, ^177^ I148M does not directly promote T2DM, obesity, or IR to affect NAFLD, although it renders the liver more sensitive to metabolic stress. The I148M variant responds to dietary changes and might be a marker for predicting the outcome of lifestyle modification treatments. The downregulation of the I148M might be a promising target for the treatment of NAFLD.

TM6SF2 plays a role in the pathway of hepatocyte VLDL secretion, which is working in the transformation of TAG into apolipoprotein B100. TM6SF2 codes a membrane protein located on the ER and ER‐Golgi intermediate compartments. Deletion of TM6SF2 in cells results in the TAG droplets accumulation, both in number and size, while overexpression of TM6SF2 reduces lipid droplets.^178^ The SNP E167K with a mutation at position 167 (rs58542926 C>T), where glutamic is substituted by a lysine, has a phenotype of high liver TAG and low circulating lipoproteins. A cohort study showed that this TM6SF2 variant is a risk factor for fibrosis in the liver and is associated with a 1.9‐fold increase in advanced fibrosis.^179^ Notably, this mutation is related to a high risk of liver disease but a low risk of cardiovascular diseases. The E167K form seems to cause a 40% reduction in cardiovascular events and a 50% reduction in atherosclerotic carotid plaques. Interestingly, individuals with a C allele (167E) have increased risks of dyslipidemia and cardiovascular disease.^180^ Further studies are urgently needed since the effect of TM6SF2 variants on the pathogenesis of NAFLD is not yet totally understood. Other genetic modifiers, such as GCKR and mitochondrial superoxide dismutase 2 (SOD2), have also been reported to influence NAFLD.^181^, ^182^

In addition to SNPs, epigenetic factors, including noncoding RNA, DNA methylation, modifications on histone proteins and chromatin remodeling, can modify the expression of genes and play important roles in the progression of NAFLD. MicroRNA (miRNA) regulates the translation and stability of message RNAs, which are the key intermediators of gene expressions. miR‐122 is one of the most attractive miRNAs related to liver pathophysiology. The inhibition of miR‐122 in a diet‐induced NAFLD mouse model can decrease cholesterol levels and increase fatty acid oxidation in the liver. To the opposite, miR‐122 overexpression leads to the progression of inflammation, fibrosis, and HCC.^98^ miR‐21, miR‐195, miR‐216a‐miR‐217 cluster, miR‐199a/b‐3p, and miR‐140‐5p are all useful epigenetic factors related to the progression of NAFLD. DNA methylation and chromatin remodeling can also regulate gene expression, affecting the progression of NAFLD.^174^ Methylation patterns have been found to act on the HSC activation‐related gene, which protects against fibrosis.^183^ Methylation of cytosine at the CpG‐rich region can suppress gene transcription and modifications of histones, including acetylation and methylation, and influence chromatin structure and function.^20^, ^184^ Moreover, trained immunity, which is orchestrated by epigenetic reprogramming and not associated with any permanent genetic modifications, is a fundamental feature of the immune response in mammals.^184^, ^185^ Compared with classical adaptive immune memory, memory within trained immunity is shorter in duration. To investigate trained immunity might bring new insights into the therapeutic strategies developments of NAFLD. Epigenetic modifications are believed to be a key determinant of NAFLD and provide new possibilities for promoting the morbidity and mortality of HCC, although available data remain in their infancy. More comprehensive studies are needed regarding the application of epigenetic factors to the diagnostic and therapeutic goals of NAFLD.

## 6 Treatment

Although great achievements have been made in the pathogenesis of NAFLD, effective medications are still in development. Lifestyle modification remains the most recommended and effective way to improve NAFLD currently.^186^ In addition, bariatric surgery could be a treatment option for those morbidly obese NAFLD patients whereas liver transplantation is available for end‐stage NAFLD patients.

### 6.1 Lifestyle Modification

#### 6.1.1 Weight Control

Management of overall fitness and weight is the main treatment strategy for NAFLD patients, and it has been approved that a certain amount of weight loss can reduce steatosis and fibrosis.^187^ ≈90% of the patients who achieved 10% body weight loss showed obvious improvements in fibrosis, although the sustained change was difficult to achieve; more than 60% of the 7–9.99% weight loss group showed fibrosis resolution, as did 26% of the 5–6.99% weight loss group.^188^

#### 6.1.2 Dietary Supplements

Based on the discussion in Section 5, high fat, fructose, and cholesterol can all increase the risk of NAFLD. To the opposite, a diet with limited carbohydrates, a Mediterranean diet which is rich in fiber and polyunsaturated fatty acids, has been proven to reduce hepatic steatosis effectively, which result in reducing liver fat and improve insulin resistance.^154^, ^189^ ω‐3 fatty acids are a type of polyunsaturated fatty acid with antisteatosis genesis, antioxidant and anti‐inflammatory characteristics, while ω‐6 fatty acids have contrary properties. An ω‐6 fatty acid diet will induce steatosis and IR by increasing lipogenesis and decreasing fatty acid oxidation, whereas ω‐3 fatty acid supplementation in the diet could reduce the risk of NAFLD. Fish, vegetables, and other ω‐3 fatty acid–rich vegetables are recommended for patients with cardiovascular disease.^151^

Substantial changes in diet over the long term are quite difficult for these individuals. Whether a short‐term diet could promote the disease progression and fibrosis is not clear.

#### 6.1.3 Exercise

In addition to diet changes, exercise has shown great benefit for NAFLD progression. However, the optimal type, duration, intensity, and frequency of exercise remain to be defined.^190^, ^191^ It has been reported that aerobic exercise is more effective than nonaerobic exercise in reducing the intrahepatic fat which is independent of weight loss.^189^, ^191^ Similarly, high‐intensity activity shows better effects but is not suitable for all patients, especially patients with cardiovascular complications.^187^, ^192^ The most advanced strategy is a combination of diet and exercise, which might have a better effect in the long run.^189^ Furthermore, eating nocturnally, depression, and sedentary working the whole day are all risk factors for NAFLD and may be good openings for lifestyle modifications.^187^

### 6.2 Pharmacology

Effective medications for NAFLD, which are more direct and effective, are priorities because of the low feasibility of lifestyle modification.^193^ Ideally, the drugs should not damage extrahepatic organs, should have low side effects, and should benefits NAFLD.^194^ The majority of drugs in clinical trials are in or beyond phase II and the first drug is predicted to enter the market in 2020 or 2021.^195^ Herein, the most promising drugs in or beyond phase II clinical trials and promising drug targets are discussed and classified by mechanism.

#### 6.2.1 Metabolism

Countless enzymes are intertwined in the intracellular metabolism and lipotoxicity processes. Fatty acid synthase (FAS) and acetyl‐CoA carboxylase (ACC) are the key mediators in the DNL process. The transcription factors SREBP‐1c and carbohydrate‐responsive element‐binding protein (ChREBP) are in turn controlled by liver X receptors (LXRs) and can regulate the farnesoid X receptors (FXRs).^21^ Peroxisome proliferator‐activated receptors (PPARs) enhance the oxidation of lipids and the expression of fatty acid transport protein (FATPs). All of these molecules could be considered to be potential targets for the treatment of NAFLD. Furthermore, drugs used to treat T2DM and obesity, such as metformin and liraglutide, are employed to cure NAFLD because of the similarities in pathogenesis between the diseases.

*ACC Inhibitor*: A recent article reported the effects of GS 0976 in the NAFLD patients. GS 0976 is an ACC inhibitor which can reduce TAG genesis in the hepatocytes. A phase II, randomized and placebo‐controlled trial showed that GS 0976 performed well in decreasing the fibrosis marker TIMPI as well as steatosis, revealing its potential for the treatment of NAFLD.^196^

*DGAT2 Inhibitor*: DAG O‐acyltransferase (DGAT), located on the ER membrane, is involved in the final step of TAG synthesis. There are two isoforms of DGAT encoding distinct genes. DGAT2, commonly expressed in the liver and adipose tissue, plays a role in lipid metabolism in these tissues, whereas DGAT1 is expressed in the skin, skeletal muscle and intestine.^197^ Thus, inhibitors of DGAT2 could be used to reduce liver TG levels and improve steatosis, hypertriglyceridemia and IR.^27^ Vitamin B3 is an outstanding DGAT2 inhibitor used to treat oxidative stress in NAFLD, but it is limited by its side effects of the prostaglandin‐mediated flushing. Employing an advanced control release system, such as nanoparticles, to deliver the vitamin B3 could decrease its side effects and maintain the drug efficacy. Mangiferin is another recently identified DGAT2 inhibitor. This compound is always found in fruits/herbal medicines and could also be used for NAFLD.^198^

*FXR Agonists*: FXR, a nuclear hormone receptor, is activated after binding to BAs or synthetic ligands, resulting in various metabolic effects, such as insulin sensitivity in the muscle/adipose tissue and glycogenolysis in the liver.^25^ The microbiome modulates the gut‐liver axis via FXR signaling or gut‐derived hormones regulate BA synthesis, influencing the lipid and glucose homeostasis.^147^ FXR agonists can naturally be used as pharmaceuticals for NAFLD to combat inflammation and fibrosis. Obeticholic acid, a derivative of chenodeoxycholic acid, is the most promising FXR agonist used in NAFLD, and it is in phase III clinical trials.^199^ Modest weight loss and reduced levels of ALT and TG in the liver can be observed in the patients treated with obeticholic acid.

*PPAR Agonists*: PPARs, which are a type of ubiquitously expressed nuclear hormone receptor, are involved in the lipid and glucose homeostasis.^200^, ^201^ Three different isoforms of PPARs have been identified in humans, and they mediate different pathways in NAFLD: PPARγ, PPARα and PPARδ. PPARγ can help to reduce lipogenesis in hepatocytes and improve adipose tissue IR, PPARα is thought to increase the oxidation of fatty acids and the activation of PPARδ leads to increased fatty acid oxidation and decreased lipogenesis by mediating SREBP‐1c in the liver and improving inflammation. PPAR agonists seem to be effective targets for NAFLD.

Pioglitazone is a well‐studied PPARγ agonists, and it can promote insulin sensitivity and enhance lipid uptake and synthesis in adipocytes, resulting in a decrease in lipids in the liver. Moreover, pioglitazone can upregulate adiponectin which can promote the oxidation of fatty acids in the liver. The largest randomized controlled trials (RCT)–PIVENS trial showed that pioglitazone could promote histological features such as reducing the ALT level, reversing IR related to inflammation and resolving steatohepatitis.^202^ This trial tested a low dose pioglitazone which was 30 mg d^−1^ for 2 years in nondiabetic NASH patients. There are some other several RCT showed the same results with the PIVENS trial though they tested with different dosage and time duration.^203^, ^204^ And there was a review listing the detail information of different pioglitazone trials.^28^ However, the long‐term adverse effects, such as weight gain, limit the long‐term and widespread use of this drug. Elafibranor (GFT505) is a liver‐targeted drug that acts as a dual PPARα and PPARδ agonist with good safety and hepato‐protective features. Human study data have shown that GFT505 could improve the insulin sensitivity in the liver, muscles and adipose tissues as well as inflammation. Furthermore, in animal models, in addition to reducing steatosis and inflammation, GFT505 preformed antifibrotic properties, suggesting its great potential in the treatment of NAFLD. Currently, a phase III RCT is underway in patients with NAFLD.^205^ It has been reported that saroglitazar which is a PPAR α/γ agonist, can induce histological improvements and reduce liver fat.^26^

*GLP‐1 Receptor Antagonists*: Given the strong link between diabetes and NAFLD, antidiabetic drugs such as glucagonlike peptide 1 (GLP‐1) receptor antagonists and dipeptidyl peptidase‐4 (DPP‐4) inhibitors are emerging as therapeutic targets in NAFLD. GLP‐1 receptor antagonists play essential roles in glucose and lipid metabolism, and they can reduce glucagon, decrease appetite, and increase insulin secretion and activity in the liver and adipose tissue. Liraglutide is one of the best‐known GLP‐1 receptor antagonists which is used to treat T2DM. Liraglutide can significantly improve ALT levels, steatosis, and ballooning, but not inflammation, nor can it induce weight loss.^9^, ^206^ Sitagliptin, a DPP‐4 blocker, has also been observed to reduce the TG levels in the liver and ALT levels in serum.^207^ However, randomized, controlled trials with large samples are needed to draw therapeutic conclusions regarding the use of the above drugs for NAFLD.

*THR‐β Agonist*: The thyroid hormone receptor beta (THR‐β) is a type of liver thyroxine receptor which mediate the cholesterol metabolism via bile.^208^ Thus, it's believed THR‐β could be a potential drug target for NAFLD involved with the DNL progress.^79^ MGL‐3196 is a selective THR‐β agonist which have been proved to reduce liver steatosis in animal experiments.^209^ The phase II trial results showed that MGL‐3196 can significantly decrease hepatic fat in NASH patients.^210^ Other THR‐β agonist, such as VK2809 also perform abilities to decrease liver fat and increase FA oxidation and now is in a phase II clinical trial.^195^

*Metformin*: Metformin, which is used as a T2DM medication, has been described as a promising medication for NAFLD, improving steatosis and inflammation in the liver in both animal models and clinical trials.^211^ However, it inducted no improvements in liver histology and could not reverse NASH and fibrosis. Some studies have shown that metformin suppressed proliferation and tumorigenesis in hepatoma cells, resulting in the inhibition of HCC.^212^ However, according to some guidance, metformin is not recommended for adult patients.^3^

*Statins*: Statins are known as a type of lipid‐lowering drug medication whose mechanism is acting as an HMG‐CoA reductase inhibitor.^195^ Furthermore, statins have been found that they can effective improve cardiovascular disease at early stage.^213^ Since lipid accumulation is closely associated with the progression of NAFLD and cardiovascular diseases are the main mortality causes of NAFLD, statins are potential agents for the treatment of NAFLD. However, there is no evidence that can prove that statins have a benefit on liver histology in NASH patients.^214^ In some guideline, statins are not recommended for the treatment of NASH.^49^

#### 6.2.2 Gut

Since the gut is the main organ for the absorption of nutrients, gut and gut‐related targets could be promising for NAFLD treatments. Orlistat, which is an antiobesity agent, acts as a gut lipase inhibitor, decreasing the absorption of dietary lipids.^215^ Furthermore, the newly developed IMM‐124e, which acts on the gut microbiome, has undergone a phase II clinical trial and proven to be effective for NAFLD.^195^

#### 6.2.3 Oxidative Stress

Oxidative stress in hepatocytes is a characteristic of lipotoxicity thus antioxidation agents can be used to treat NAFLD. Vitamin E, which includes tocotrienol homologues and tocopherol (a widely used form), can be used in NAFLD due to its antioxidation ability. Study data from animal models have indicated that vitamin E can protect hepatocytes from injury by combating mitochondrial toxicity. The PIVENS trial showed that vitamin E treatment could significantly improve the steatosis, ballooning, inflammation and histological features.^202^ In the TONIC trial, which was conducted in pediatric patients, vitamin E failed to meet the end point, although in some guidelines, vitamin E is suggested for treating NAFLD.^78^, ^216^, ^217^, ^218^ Larger‐scale trials are needed to define the effects of vitamin E in NAFLD and its extension to clinical application. The lack of composition or packaging standards for vitamin E and the pro‐oxidant effect caused by high concentrations of vitamin E limit its widespread use. Pentoxifylline, a nonspecific phosphodiesterase inhibitor, is used to target peripheral vascular disease. The use of pentoxifylline in NAFLD can lead to reduced inflammation and fibrosis via the cyclic AMP pathway. Studies with animal models have shown that pentoxifylline suppressed cytokine release and had antioxidant effects; however, data from large population samples are lacking. A short‐term trial in 55 patients revealed that pentoxifylline had good antioxidation effects.^219^, ^220^ Natural antioxidative agents, including resveratrol, curcumin, and sulforaphane have also been studied, and several clinical trials are underway.^26^

#### 6.2.4 Inflammation, Cell Injury, and Cell Death

Inflammation occurs in NASH, inducing both innate and adaptive immune responses. Targets involved in proinflammatory pathways can be considered in treatments for NAFLD.

*CCR Receptor Antagonist*: In hepatocyte injury progression, C‐C chemokine ligand type 2 (CCL2), secreted by Kupffer cells, can active CCR2 receptors, leading to an inflammatory reaction.^158^ Cenicriviroc (CVC), a dual CCR2/CCR5 receptor antagonist, proved to be important for inflammation and fibrosis progression in the liver. Phase IIb trial results showed that CVC could significantly improve fibrosis compared to placebo.^221^

*ASK1 Inhibitors*: ASK1, also known as MAP3K5, is an important player in the progression of NAFLD. The hyperactivation of ASK1 in the liver is commonly observed in NAFLD patients, and ASK1 inhibitor could be a promising target for the treatment of NAFLD, although the molecular mechanism is nascent. ASK1, which can be activated by ROS, TNFα, LPS, and ER stress, is implicated in the cell death and fibrosis and promotes lipid/glucose metabolism disorders and inflammation in the liver via the downstream p38/JNK pathway.^121^, ^123^, ^129^, ^222^ Selonsertib (GS‐4997) is a small molecular ASK1 inhibitor for the treatment of NAFLD. Phase IIb and III trial results showed that selonsertib could improve the bridging fibrosis and cirrhosis in NAFLD patients.^223^ However, selonsertib also showed daunting side effects because it acted as an ATP competitor which could entirely inhibit ASK1 activity. Thus, it is important to determine the mechanism of ASK1 in NAFLD for the purpose of developing novel targets in this pathway which could keep the normal function of ASK1. As discussed in Section 5, our lab has made some progress in ASK1‐related pathways, bringing new insights into the identification of smart ASK1 inhibitors.

*TAK1 Inhibitor*: Similar to ASK1, TAK1, a type of upstream MAPK pathway kinase, is a central mediator in the progression of NASH, which involves metabolic disturbance, inflammation and cell death.^126^, ^128^, ^131^ TAK1 has been determined to be a regulator in both the IKK/NF‐κB and JNK pathways, which makes it crucial for the regulation of inflammation related genes. Furthermore, hyperactivation of TAK1 caused by metabolic stress induces metabolic disorders and NASH via the NF‐κB and JNK/p38 pathways.^103^ Thus, the suppression of TAK1 activation is a potential strategy to treat NAFLD, although it is still in preclinical phase. It has been reported that an adequate amount of CYLD impairs the progression of NASH by inhibiting TAK1 signaling, and the E3 ligase TRIM47 has been revealed to be a key regulator of CYLD degradation, revealing that both of the above targets could be significant for the treatment of NAFLD.^92^

*Caspase Inhibitor*: Emricasan is a caspase inhibitor which can reduce the portal hypertension via blocking the activation of inflammatory caspase and inhibiting hepatocyte cell death. A phase IIa study showed that emricasan caused obvious decreases in AST and ALT levels, whereas a phase IIb study proved that emricasan could improve fibrosis in NASH patients.^224^

#### 6.2.5 Fibrogenesis

Reducing fibrosis is a key goal of NAFLD therapy.

*Galectin‐3 Inhibitor*: Galectin‐3 is a key protein in the fibrogenesis process, which is expressed by immune cells. GR‐MD‐02 was developed as a galectin‐3 inhibitor for use in NAFLD with the aim of suppressing fibrogenesis progression. The antifibrosis characteristics of GR‐MD‐02 have been confirmed in animal models, and human clinical trials are underway.

*LOXL2 Inhibitor*: Lysil oxidase homologue 2 (LOXL2) is an enzyme associated with ECM formation, which is a key process in fibrogenesis.^225^ Simutuzumab was developed as an effective anti‐LOXL2 monoclonal antibody, with a long half‐life and was shown to reduce activated fibroblasts, growth factors and cytokine expression.^226^ Simutuzumab is currently used for various solid tumors, and trials in NAFLD in patients with NASH and even advanced fibrosis or cirrhosis are underway.

*Leukotriene Receptor Antagonist*: MN‐001, which has shown potent anti‐inflammatory and antifibrogenesis abilities, is now being used to treat NAFLD. MN‐001 is involved in several progressive processes, such as leukotriene receptor antagonist, phosphodiesterase 3 and 5‐LO inhibition. In animal models, MN‐001 could suppress inflammation and fibrosis and reduce the expression of proinflammatory and profibrogenic genes, such as CCR2 and LOXL2.^227^ A phase IIb randomized trial is underway. **Table** **4** lists detailed information about some of the promising drugs in/beyond phase II.

**Table 4:** Promising medications for NAFLD. NIDDK, National Institute of Diabetes and Digestive and Kidney Diseases; FXR, farnesoid X receptors; PPAR, peroxisome proliferator‐activated receptors; SCD‐1, stearoyl‐CoA desaturase‐1; ACC, acetyl‐CoA carboxylase; GLP, glucagonlike peptide; LPS, lipopolysaccharide; PDE, phosphodiesterase; CCR, C‐C chemokine receptor; ASK1, apoptosis signal‐regulating kinase 1; LOXL2, lysil oxidase homologue 2

Drugs	Developer	Mechanism	Highest phase	Clinical trial number	Primary outcomes & limitations
***Metabolism and Lipitoxicity***					
INT‐747 (Obeticholic acid)	Intercept Pharmaceuticals	FXR agonist	Phase III	NCT02548351	Fibrosis improvement, side effect, pruritic
Pioglitatone	NIDDK	PPAR γ agonist	Phase III	NCT00063622	Improvement of steatosis, ballooning and inflammation, weight loss
GFT 505 (Elafibranor)	Genfit	PPAR α/σ agonist	Phase III	NCT02704403	Resolution of NASH without worsening fibrosis
saroglitazar	Zydus Discovery	PPAR α/γ agonist	Phase III	NCT03061721	Change in ALT level
Aramchol	Galmed Pharmaceuticals	SCD‐1 inhibitor	Phase III	NCT02279524	Improvement in fat
LJN 452	Novartis Pharmaceuticals	FXR agonist	Phase II	NCT02855164	Change in transaminases
GS 0976	Gilead Science	ACC inhibitor	Phase II	NCT02856555	Safety, efficacy, tolerability
MGL‐3196	Madrigal Pharmaceuticals	THR‐β agonist	Phase II	NCT02912260	Improvement in liver fat
Metformin	NIDDK	/	Phase III	NCT00063635	No improvement on histological, gastrointestinal side effect
Liraglutide	Novo Nordisk	GLP‐1 agonists	Phase II	NCT01237119	Improve IR and ALT level
***Gut***					
orlistat	/	Intestinal lipase inhibitor	Phase II	NCT00160407	Improvement in liver fat
IMM 124e	Immuron	Anti‐LPS	Phase II	NCT02316717	Improvement in liver fat
***Oxidation***					
Vitamin E	/	Antioxidation	Phase III	NCT00063635	Improvement in liver fat
pentoxifyline	/	PDE inhibitor	Phase II	NCT00590161	Improvement in liver fat
***Inflammation***					
TBR‐652 (Cenicriviroc)	Allergan	CCR2/5 antagonist	Phase II	NCT03028740	Fibrosis improvement
***Cell death***					
GS 4997 (Selonsertib)	Gilead Science	ASK1 inhibitor	Phase III	NCT03053050	Fibrosis improvement
IDN 6556 (Emricasan)	Conatus Pharmaceuticals	Caspase inbibitor	Phase II	NCT02686762	Fibrosis improvement
***Fibrosis***					
GR‐MD‐02	Galectin Therapeutics	Galectin 3 inhibitor	Phase II	NCT02462967	Improvement in HVPG
GS 6624 (Simtuzumab)	Gilead Science	LOXL2 inhibitor	Phase IIb	NCT01672866	Fibrosis improvement
MN‐001 (tipelukast)	MediciNova	Leukotriene receptor antagonist	Phase II	NCT02681055	Decreasing TAG level

Effective medications for NAFLD are strongly demanded, as are novel specific therapeutic targets. Due to the complex pathophysiology of NAFLD, combination therapies acting on more than a single target might be a promising way to provide synergistic histological benefits.

### 6.3 Bariatric Surgery and Liver Transplantation

Besides lifestyle modifications and pharmacological strategies, surgery can be an effective way to treat urgent NAFLD patients who are at risk of obesity‐ or HCC‐related co‐mortalities, including cardiovascular diseases and malignancy. Bariatric surgery and liver transplantation are alternative options for NAFLD patients.

Bariatric surgery is an excellent method for helping obese patients lose weight and reducing the risk of cardiovascular disease‐related comorbidities. Patients with a BMI > 35 kg m^−2^ and severe comorbid factors, such as cardiomyopathy and hypertension, are suggested to undergo bariatric surgery. Many studies have revealed that bariatric surgery can improve or even reverse steatosis, NASH and fibrosis, and the improvement extends beyond weight loss. Gastric bypass is one of the most mature bariatric surgeries.^228^ However, the complications and the inescapable risks of this invasive surgery limit its further application for the general patients.

At present, NASH is the second leading indication for the liver transplantation (LT) after alcoholic liver disease and is the prominent reason for LT in women.^229^, ^230^, ^231^ Leaving the possibility of transplant rejection aside, the transplanted liver cannot be protected from the risk of NAFLD, and its high cost and the low availability of suitable liver resources limit the application of LT.

In summary, conquering this highly prevalent disease remains a considerable challenge, and no effort should be spared in the fight against NAFLD, including early lifestyle interventions and multidisciplinary management.

## 7 Perspective

NAFLD is currently a prominent chronic liver disease worldwide. No effective interventions are currently available, placing a tremendous burden on the health of global populations. The current guidelines and guidance for NAFLD are heterogeneous and fragmentary, revealing an urgent need for authoritative standards regarding the screening, diagnosis and management of NAFLD and an appeal emphasizing the need for awareness of and caution of regarding this epidemic disease by governments worldwide. Increased resources should be devoted to the investigation of NAFLD, especially its pathogenesis and potential therapeutics, as well as effective screening and diagnosis tools. The development of animal models, especially nonhuman primate models, can accelerate the translation from basic research to clinical application, which is also imperative and require further study. The etiologies of NAFLD are complex, implicated with a myriad of factors, and the driving forces in different phases of disease and in various patients are discrepant, which increases the difficulty of treatment. A multiomic approach can be a good choice for better understanding of the pathogenesis of NAFLD, and precise or combined treatments seems to offer more promising options for conquering this disease. It is not too late to fight against NAFLD. The public, governments, clinicians, researchers and industries should unite together to impair the prevalence of this daunting disease.

## Conflict of Interest

The authors declare no conflict of interest.
